# “It beats the hell out of going to a hospital”: service user experiences of telemedicine-based symptom-triggered alcohol withdrawal management

**DOI:** 10.1186/s13722-025-00585-8

**Published:** 2025-08-13

**Authors:** Nikki Bozinoff, Divya Prasad, Ke Bin Xiao, Anthony Ngoy, Bernard Le Foll, Anna Gordezky, Christian S. Hendershot, Sandra LaFleur, Lena C. Quilty, Victor M. Tang, Tara Marie Watson, Matthew E. Sloan

**Affiliations:** 1 Addictions Division, Centre for Addiction and Mental Health, Toronto, ON Canada; 2 Department of Family and Community Medicine, University of Toronto, Toronto, ON Canada; 3 Campbell Family Mental Health Research Institute, Centre for Addiction and Mental Health, Toronto, ON Canada; 4 Department of Psychiatry, Warneford Hospital, University of Oxford, Oxford, UK; 5 Institute for Mental Health Policy Research, Centre for Addiction and Mental Health, Toronto, ON Canada; 6 Department of Psychiatry, University of Toronto, Toronto, ON Canada; 7 Department of Pharmacology & Toxicology, University of Toronto, Toronto, ON Canada; 8 Institute of Medical Science, Temerty Faculty of Medicine, University of Toronto, Toronto, ON Canada; 9 Translational Addiction Research Laboratory, Centre for Addiction and Mental Health, Toronto, ON Canada; 10 Waypoint Research Institute, Waypoint Centre for Mental Health Care, Penetanguishene, ON Canada; 11 Remote Alcohol Withdrawal Community Advisory Board, Centre for Addiction and Mental Health, Toronto, ON Canada; 12 Department of Population and Public Health Sciences and Institute for Addiction Science, Keck School of Medicine, University of Southern California, Los Angeles, USA; 13 Department of Psychological Clinical Science, University of Toronto Scarborough, Toronto, ON Canada

**Keywords:** Alcohol withdrawal, Patient perspectives, Qualitative, Telemedicine, Remote

## Abstract

**Introduction:**

Increasingly, services for the management of substance use disorders have been developed or adapted for remote delivery. Limited research has investigated service user experience of these services. We undertook a qualitative sub-study, embedded within a pilot feasibility study of remote symptom-triggered alcohol withdrawal management, to better understand the experiences of participants. Our aim was to determine the acceptability of the intervention and refine intervention procedures.

**Methods:**

Eligible participants were enrolled in the parent study and completed at least one day of telemedicine-delivered symptom-triggered alcohol withdrawal management. Individuals were adults with alcohol use disorder recruited using intensity sampling. Participants completed an audio-recorded, semi-structured interview. Thematic analysis was conducted using Braun and Clarke interpretive methodology.

**Results:**

Fourteen individuals were enrolled in the study. Six themes were identified: benefits of being in the home environment, technological tensions, intervention-specific feedback, personal motivations for participation, post-program achievements and changes and navigating the ‘system’. Participants identified numerous benefits of being in the home environment including: increased comfort, privacy and security, normalizing abstinence in the home, flexibility to engage in other tasks, and the convenience of not travelling. Intervention-specific feedback included positive aspects of the intervention (interactions with staff, accountability, counselling, use of medication), areas for improvement (preparation, scheduling, medication logistics, and aftercare), and the meaning and role of having a support person available during treatment.

**Conclusion:**

Participants found remote alcohol withdrawal management to be satisfactory and associated with several benefits including increased comfort, privacy, normalizing abstinence in the home, flexibility and convenience. They also provided important feedback for refinement of the intervention. Findings suggest that remote alcohol withdrawal management could play an important role in improving access to medical management of alcohol withdrawal, particularly in rural, remote or underserved areas.

**Supplementary Information:**

The online version contains supplementary material available at 10.1186/s13722-025-00585-8.

## Introduction

As a result of the COVID-19 pandemic, many services for the management of substance use disorders were developed or adapted for remote delivery. Rather than being a transient phenomenon, the shift toward telemedicine-based mental health services has persisted as their benefits for patient-centered care have become more evident; multiple reviews, for example, suggest that telemedicine interventions for alcohol use disorder (AUD) are effective [1–3], with positive outcomes such as reduced substance use [1, 3, 4] and improved treatment retention [1, 4, 5]. However, limited research has focused on service user experiences [1, 6–9] which is necessary to refine novel interventions.

The first step in AUD treatment often involves managing alcohol withdrawal syndrome (AWS). Symptom-triggered alcohol withdrawal management using a symptom severity scale such as the Clinical Institute Withdrawal Assessment of Alcohol Scale, Revised (CIWA-Ar) is often preferred over fixed-dose benzodiazepine management due to reduced treatment duration and lower amounts of benzodiazepines required [10]. In a recent pilot clinical trial, we sought to determine whether symptom-triggered alcohol withdrawal management could be feasibly delivered over telemedicine [11]. The pilot clinical trial enrolled 30 actively drinking participants with AUD and a history of alcohol withdrawal and found > 90% completed all three days of the remote symptom-triggered withdrawal management procedure and none required transfer to a higher level of care [11]. The expansion of this novel intervention could have several advantages including improved convenience and the ability to provide withdrawal management in remote settings that lack specialized services.

Integration of qualitative research within clinical trials in addiction medicine has increasingly been recognized as important and can provide valuable information about intervention acceptability, theory of outcomes achieved, and inform improvements to the intervention [12]. Here we report results from a qualitative sub-study that explored the perceptions of care received by service users and the challenges and benefits associated with the use of telemedicine to treat AWS. Specifically, we aimed to understand acceptability and inform the refinement of the intervention, which has the potential to significantly improve access to medical alcohol withdrawal management.

## Methods

### Study design and setting

This qualitative sub-study was embedded within a pilot clinical trial that aimed to assess the feasibility of delivering symptom-triggered alcohol withdrawal management via telemedicine. The primary outcomes of the parent study were retention in treatment and safety (i.e., need for transfer to a higher level of care such as the emergency room). The intervention has been described in detail previously [11] (https://clinicaltrials.gov/study/NCT04858490). In brief, participants were primarily recruited from a tertiary care outpatient addiction clinic specializing in longitudinal management of substance use disorders and concurrent mental illness in Canada (Centre for Addiction and Mental Health [CAMH]). In this clinical setting, standard of care for alcohol withdrawal may include the following, depending on symptom severity, clinician judgement, and patient-preference: fixed-dose home withdrawal management, ‘day detox’ (an in-person ambulatory service for symptom-triggered withdrawal where the individuals return home overnight), admission to an inpatient medical withdrawal service, or withdrawal management via the emergency department. Eligible participants were *≥* 18 years, actively using alcohol, and met DSM-5 criteria B for AWS. Exclusions included a history of complicated AWS (i.e. seizures, hallucinations, delirium tremens); severe medical or psychiatric co-morbidity that would prevent safe participation in the study, and those without stable housing. In advance of their participation, study staff provided a presentation and detailed document to the participants about what to do if they experienced any complicated withdrawal symptoms or adverse effects. Participants had an opportunity to test the telemedicine platform and were provided a study tablet if they did not have access to a device with internet connection and a webcam. No potential participants in the parent study declined participation at the time of pre-screening or eligibility assessment due to technological barriers. An initial prescription for diazepam (typically 80 mg) was written for the client to pick up prior to remote alcohol withdrawal management. A trained nurse or physician used a modified version of the CIWA-Ar adapted to assess withdrawal severity over the telemedicine platform. If CIWA-Ar score was *≥* 10, participants were advised to take diazepam 10 or 20 mg at the treating physician’s discretion and reassessed every hour. When CIWA scores were < 10, the participant was reassessed every 1–4 h and diazapem would be started only if subsequent scores were above 10. Monitoring lasted 3 days but could be extended by 1-2 days if withdrawal symptoms were not resolved by the end of day 3. Following completion of withdrawal management, participants were offered treatment with medications for alcohol use disorder (MAUD) and weekly virtual cognitive behavioural therapy for four weeks.

### Participants

For this qualitative sub-study, eligible participants were those who enrolled in the parent study who had participated in at least one day of the remote procedure. Participants dropping out of treatment or withdrawing from the main study were eligible for participation provided they met the above criteria. All participants provided informed consent prior to participation. The study was approved by the CAMH Research Ethics Board.

### Recruitment and sampling

As we were interested in understanding service user perceptions of care, we used purposive sampling, specifically intensity sampling, to identify information rich cases [13]. We relied on the parent study nurse and research coordinator to identify potentially information-rich cases. Specifically, they were asked to identify potential participants who voiced opinions related to their care, the intervention, or experience with telemedicine technology (positive or negative). Additionally, our protocol specified that any persons dropping out of the intervention prior to day 3, or those being transferred to a higher level of care during the course of treatment would be contacted to participate once medically stable (criterion sampling) [13], as they were assumed to be information-rich cases. Participants were contacted either by phone or by email following day 3 of the intervention (or after treatment discontinuation if they did not complete treatment) and invited for a telephone or video interview. Recruitment continued until meaning saturation was reached, that is, the point when the research team perceived they fully understood the meaning of codes and themes, and additional interviews yielded no further dimensions, nuances or insights [14].

### Data collection

Demographic information was collected from participants, including age, gender, race, and employment status. Semi-structured interviews were conducted by a trained Master’s level research assistant (DP) who was not involved in the clinical care of the participants, using an interview guide developed by the research team in consultation with the Community Advisory Board (Appendix 1). The interview guide was designed to use broad, open-ended questions to gain in-depth understanding of the care experience. Interviews were conducted via Webex platform, audio-recorded, transcribed verbatim, and back-checked for accuracy. All data files were managed in NVivo 12 software. Interviews took place between January 2022 and August 2023.

### Data analysis

An inductive thematic analysis was conducted using Braun and Clarke thematic analytic methodology [15]. Several study team members (NB, TMW, and DP) independently read and coded the first 5 interview transcripts to develop an initial coding scheme. Differences in individual coding were resolved through discussion until team consensus was achieved (triangulation). DP then coded all subsequent interviews using this initial coding scheme, adding new codes where prior codes did not capture emerging ideas. The research team met again after every 3–5 new interviews to review and refine the coding scheme in view of additional codes. Once all data were coded, the team sorted codes into potential overarching themes and sub-themes by developing a thematic map document, which also included key quotes and excerpts that illustrated each theme. The coded data extracts for each theme were then reviewed to ensure they formed a coherent idea and adjustments to themes were made as needed. Finally, the research team reviewed the entire thematic map to ensure it reflected the broad meaning of the dataset. Throughout the research process, trustworthiness was enhanced via triangulation with core research team members, a detailed audit trail, thick description of the primary study and study context, as well as involvement of members of our community advisory board in all aspects of the research process including providing input into the thematic map [16]. The consolidated criteria for reporting qualitative research was followed in reporting the study [17].

### Reflexivity statement

This qualitative study was built on our shared commitment to improve withdrawal management care for persons with AUD. The authors acknowledge the heterogeneous professional and personal identities that they bring to this work. Among the core research team for this sub-study NB, TMW, and MS all have > 10 years of experience conducting substance use research in academic settings. NB is an addiction medicine and family physician, as well as a clinician scientist. DP is currently a doctoral graduate student, and at the time of her employment had completed her MSc. MS is an addiction psychiatrist and the Principal Investigator for the parent study. TMW holds a PhD in criminology and is an academic research program administrator. The project received input from a multidisciplinary team of clinicians and a community advisory board composed of persons with lived/living experience of AUD and family members.

## Results

Fourteen individuals were interviewed. The median interview duration was 38 min (Interquartile Range [IQR]: 29–46 min) and the median time from remote alcohol withdrawal management to the interview was 51 days [IQR: 19–70] The median age was 46 (IQR:39–52). 9/14 (64.3%) identified as men and 11/14 (78.6%) identified as White. In terms of education, 5/14 (35.7%) participants had completed a university or other post-graduate degree, 4/14 (28.6) had completed college or other non-university certificates or diplomas, and 5/14 (35.7%) completed high school or less. Eight out of 14 (57.1%) reported being employed.

There were six overarching themes: Benefits of the home environment, technological tensions, intervention-related feedback, personal motivations for participation, post-program achievements and changes, and navigating the ‘system’.

### Benefits of the home environment

Within this theme, participants discussed aspects of being home while receiving medical support. Often they compared their experience in the study to past experiences of withdrawal management. Benefits of the home environment included increased comfort, privacy and security, flexibility to engage in other activities, the convenience of not travelling, and normalizing abstinence in the home.

Repeatedly, the concept of increased ‘comfort’ was mentioned:

“I did go through the facility [hospital] and I saw where the day detox was. And, it’s a bit of a stark environment. It’s a bit dark and drabby, you know? It’s very institutionalized, at that place. So when I’m going through my withdrawal, I’m just doing the withdrawal in an environment in which I’m already comfortable with, in a place that I already call home.” (Participant 6, Male, 38 years, White).

“It allowed me to feel a little more comfortable and at ease…I knew where I was, and all that jazz.” (Participant 14, Male, 52 years, White).

Other benefits of the home environment included increased privacy and security:

“Been there, done it, and it’s much nicer to be in your own environment. It’s more relaxing than [elsewhere where] you’re in a room with three or four other people… I liked it remote ‘cause you have your own privacy.” (Participant 4, Woman, 62 years, White).

“You’re in the day detox room, and there’s a bunch of other people in there, right? And it’s just nicer to, nicer to have it be your own thing, versus having to kind of experience it with other people behind, like, behind the curtain.” (Participant 7, Female, 41 years, White).

Participants noted that being in the home environment acted to normalize abstinence in their usual environment, which increased their confidence and supported their recovery:

“For me, it built that confidence, and that was always my fear, and I think people’s fear in general when they go through detox, like, ‘What is going to happen? Am I going to get through this? And ‘oh my god, I’m not gonna have a drink today,’ and all that stuff… I already knew what that would be like. So it was sort of an extra confidence booster for me, to be able to do it from home, knowing that I could do it on my own.” (Participant 5, Man, 52 years, White).

“When you’re able to associate sobriety with your everyday behaviours, environment and your…daily routine. When you’re able to combine those and work on your daily routine and not have alcohol and, you know, build a new daily routine in your own environment, I think that’s really beneficial.” (Participant 2, Woman, 34 years, White).

Participants also frequently described the benefits of flexibility associated with remote withdrawal management highlighting other activities they could do while participating in remote withdrawal management that might not be possible in traditional care environments. This included having access to distractions such as entertainment, preparing food, or doing chores around the house:

“I don’t know what people do for eight hours down at the actual hospital thing, but at least I had stuff to keep me occupied, distracted. I could go for a walk, go for a bike ride and do my laundry if I had to.” (Participant 8, Man, 33 years, White).

“It beats the hell out of going to a hospital…You get to stay home and do what you do. You know, read your books, listen to your talking books, watch TV, have naps. You know? It’s completely different to be able to stay home.” (Participant 9, Man, 72 years, White).

Additionally, several participants noted that remote withdrawal afforded them flexibility that minimized disruptions to their employment:

“The remote aspect really worked for me, because I am working full time, and so it gave me the opportunity to be able to do the study, but also work and like, live my life simultaneously, so that there were no interruptions.” (Participant 2, Woman, 34 years, White).

Finally, participants noted the convenience of not having to travel to access medical care:

“I think it should be the standard now. It’s way convenient…I mean, it’s just annoying me that you go to sit at a doctor’s office, and you wait for two hours, pay twenty five dollars for parking… People go to the doctor for so many stressful reasons. Why, all these layers of stress, for travel, for money, why add to it?… This is very convenient, and easy.” (Participant 10, Woman, 44 years, White).

### Technological tensions

This theme coalesced around perspectives related to the benefits and drawbacks of the use of technology. Most participants reported not having difficulty with the technology:

“It was pretty straightforward. Even for the, you know, computer illiterate like myself… it was pretty good. Pretty straightforward.” (Participant 14, Man, 52, White).

Some participants however, reported needing support to ensure the technology was functioning which were typically transient problems that were solved with support:

“It technically didn’t work on my phone, so they made me go into [hospital], which is a drag, cause I live in [area redacted]. So they would lend me this tablet, and showed me how to use it… the first challenge was the technology, which was a bumpy road for a couple of days.” (Participant 9, Man, 72 years, White).

"Other challenges with respect to technology were concerns from participants that they had difficulty expressing themselves to study staff via videothroughout the intervention procedure:

“So, I think that’s the biggest thing; it’s wanting to do it in the comfort of your own home, but also, not having that full experience of being looked at as if you were right standing in front of me…. Being able to communicate those feelings was really hard for me, because I was already going through the withdrawals, and not being able to express fully what I was feeling…. I kind of talked about, like, my sweaty hands, where they can’t just come over and touch your hands to feel it, but you have to kind of express it.” (Participant 2, Woman, 34 years, White).

### Intervention-related feedback

This theme was characterized by positive elements of the intervention, areas for improvement, and meaning and role of support persons.

Participants routinely reported that their interactions with staff were a highlight:

“The support staff was amazing. Everything there was like, I never felt alone. I never felt like ‘Oh my god.’ I didn’t have that fear, like, ‘What if something happens, or I’m not doing the right thing. There’s no one, like, right there.’ So yeah, they were great. They were there every step of the way” (Participant 5, Man, 52 years, White).

“The individuals who were helping me were very professional, knowledgeable, compassionate, efficient, responsive, available, friendly, kind.” (Participant 11, Man, 54 years, White).

Participants felt reassured by the accountability offered by the frequent check-ins for CIWA-Ar administration and planned counselling sessions:

“So, I have more frequent visits with my doctor, you know, and then the counselling doctor. All these meetings, appointments and that, helped me because of the accountability.” (Participant 1, Man, 45 years, East Asian).

“Another thing that was, I won’t say bothersome, more annoying, but a duty, was to maintain those meetings every few hours. And when you’re feeling sick and when you’ve vomited a few times and you’re exhausted; you’ve got headaches, you feel angry, all those withdrawal symptoms, all those hangover symptoms. And then to meet, the online meetings every hour or every two hours, that was challenging but necessary.” (Participant 12, Man, 50 years, White).

They voiced that the use of diazepam for symptom control and the use of psychotherapy and MAUD for relapse prevention were helpful aspects of the intervention.

Participants highlighted medication management, scheduling, preparation, and aftercare as areas for improvement. Complaints related to medication surrounded logistics of obtaining the medication at the pharmacy in advance of the remote withdrawal procedure and disposing of unused medication. Initially, the medication was only available on the morning of the remote procedure. Later in the intervention, the procedure was changed to allow early pick up of the medication as a result of this feedback:

“So I went down at like eight fifteen in the morning, to try to pick up my medication, and then it wasn’t going to be ready for another thirty plus minutes, so I had to walk back, to get on the call and then go back to the pharmacy again.” (Participant 7, Woman, 41 years, White).

“I only took three pills and then you tell me to go back to the pharmacy and make the pharmacist throw out four pills. It makes no sense. It makes no sense. What, did you think I was going to sell them online or something? It’s nonsense.” (Participant 3, Man, 47 years, Latino).

Similarly, some participants reported logistical challenges with scheduling the remote withdrawal intervention:

“It took a few days before it happened. It was like, between my [eligibility] interview and the beginning was probably approximately a week. So there was a lot of insomnia and anxiety, about starting it.” (Participant 9, Man, 72 years, White).

Service users also suggested that preparation could be improved, although they had differing opinions about what that might look like. Some participants who did not have experience of AWS management voiced that they would have wanted more information about what to expect about AWS itself. One participant noted that they would have appreciated documentation more in advance to support time off work, while another suggested that advice be provided about cutting down in advance.

Finally, some participants wished the intervention had more robust integration with aftercare:

“For the detox portion of it, withdrawal management for four days, it was like, what a luxury to have that kind of intense outpatient, very caring care…For withdrawal, it was great, but the follow up wasn’t enough for me to keep going. ”(Participant 10, Woman, 44 years, White).

“I felt really good about the experience. It was good to have consistent communication and contact with the people who are conducting the study, over those three days…I wish it was longer, just because I did go back to drinking” (Participant 2, Woman, 34 years, White).

The third intervention-related feedback sub-theme was ‘meaning and role of support persons’. Some participants reported having support persons (someone outside of the care team) in the home during the intervention. The majority found support persons to helpful. They reported support persons offered comfort, emotional support, material and organizational support, as well as an increased sense of safety:

“There’s also that fear of ‘What happens if I were to pass out?’ or something were to happen to me? I’m also going through a huge change of pace of not having alcohol in my system. So, I just don’t think I would have been able to fully do it on my own. Having that support was definitely a big help.” (Participant 2, Woman, 34 years, White).

“I had my wife here. That’s another good reason why I wanted it to be at home. My wife is very supportive and she’s here.” (Participant 11, Male, 54 years, White).

Conversely, some service users reported having arguments and increased interpersonal conflict due to the support person:

“I think I’ve gone through this, and I actually find sometimes, the support that my husband gives me is not the type of support that I want. So, in my particular situation, I didn’t want him encouraging me or saying too much. ‘Cause I find it more annoying than helpful.” (Participant 7, Woman, 41 years, White).

Those service users interviewed who did not have support persons in the home during the intervention reported that they did not think a support person was needed or that they could be potentially harmful:

“To be honest, if I had to say, for myself, I think the support person would have been more of a distraction and then maybe I would have been uncomfortable, by having them see me be uncomfortable, right? Because now they’re going through their own stuff, watching me go through this, and it creates more of a negative aura.” (Participant 6, Man, 38 years, White).

### Personal motivations for participation

This theme captured reasons clients reported being interested in the remote alcohol withdrawal intervention. These included anticipated convenience, comfort and privacy:

“Originally, I refused to do detox in the hospital, in a strange bed, amongst strangers…When I found out that I could do an in house detox, I immediately went for it.” (Participant 9, Man, 72 years, White).

Similar, but distinct from convenience, several participants anticipated that barriers to traditional withdrawal management with respect to employment would be mitigated with remote alcohol withdrawal management and some participants highlighted that they would not otherwise have accessed supervised withdrawal management:

“I cannot do inpatient because…I run my own business. If I go in inpatient…I would lose my business, and if I lose my business, I lose everything, in my mind. Like, coming out, I would have nothing to come back to. So, there would be no point, I think. So, I’m trying to maintain my livelihood, and also get well, right?… I cannot spend all day in a hospital.” Participant 10, Woman, 44 years, White)

Finally, participants commonly voiced that wanting to stop using alcohol was a significant motivator for participation in the study, although these motivations were in relation to making a change generally or undergoing withdrawal management generally, rather than specific to remote delivery of the intervention:

“Realizing that the situation had kind of escalated a bit and it was beginning to impact things at home, with my relationship with my husband and arguing, and kind of the kids seeing arguing, so that was also there as well, just a motivation to make a change.” (Participant 7, Woman, 41 years, White).

### Post-program achievements and changes

Participants generally had positive things to say about achievements and changes following the remote alcohol withdrawal intervention. These included improved connections, ongoing sobriety, improved financial situation, improved health and quality of life, and increased insight into their disease.

Participants routinely reported feeling connected to the study team:

“It’s the connection to Dr. X…because this one didn’t work out for me, but he followed up and tried to find, again, a follow-up medication to try to help me with the continued management of the craving. So we tried a medication for that, and it didn’t work out. So then, he recommended another one, and so we’re trying that and then I’m going to participate in yet another study, which is outpatient treatment again…It’s connection to, actually, to the resource, like, just maintaining contact and exploring more options in terms of treatment options that you guys are offering.” (Participant 10, Woman, 44 years, White).

A number of participants reported maintained gains which they attributed to the intervention:

“I was a little apprehensive to begin with, because I wasn’t sure, because I was drinking so much, that I did have pretty bad withdrawals. So I was a little nervous and apprehensive. But doing it, I felt very comfortable doing it, and it helped me. I haven’t had a drink since January tenth. So I’ve quit it completely.” (Participant 6, Man, 38 years, White).

Benefits also included improved finances:

“I’m saving a lot of money. You know, a bottle and a half a day, at thirteen dollars a bottle. You know, I keep checking my calculator, (laugh) how many hundred I’ve saved already. The financial part is quite welcome.” (Participant 9, Man, 72 years, White).

And, improved quality of life and health:

“It makes you feel good. And you feel better too. So, like, me, it was a world of difference. Like I went from being sick for a little over a year, not being able to function at all, like, totally not functioning. I never did any housework, laundry, cooking, cleaning, dishes, for over a year.…And now, it’s like totally different. I have a life.” (Participant 4, Woman, 62 years, White).

Participants also reported increased insight about their disease, treatment or self-awareness:

“I knew it was a habit before, for sure. But I didn’t realize how much subconsciously, my brain was going for that [alcohol], without me even kind of realizing. I walked by and you know, I remember getting off the subway, at some point, and I was like ‘Oh, there’s a Wine Rack. It’s an easy spot to go pick up some wine.’ And I was like ‘Oh wait, why would I do that? I don’t need to do that anymore.’ And it was just, it just kind of popped in there for some reason. But now I can realize that yeah, that it was just kind of stuck in my head.” (Participant 8, Man, 33 years, White)

### Navigating the ‘system’

The final theme related to participants’ past and ongoing challenges of navigating the mental health system. The mental health system was persistently described as disjointed, and participants had difficulty meeting their treatment needs in time and space, as well as treating their mental health and substance use health concurrently. Many described not understanding what services were available, or what a path for treatment of AUD ought to look like.

“I would have liked to have done this sooner. Right?… I had reached out a long time ago, but once again, you have to go through the hoops in order to figure out what’s what and/or to figure out what kind of treatment, what kind of help you need.” (Participant 6, Man 38 Years, White).

“Overall, I think that, I mean, challenges would just be navigating, like, ‘What do I do after?’ Like, I don’t know, I mean, I know CAMH can only offer so many resources, but you have to be your own advocate really, to get what you want done and to like, really motivate you, unless you pay, like, fifty thousand dollars and actually go to rehab. You know? It’s, it’s been an interesting experience working with our public healthcare system, when you have substance abuse and mental health issues, because it’s a challenge to navigate it and to get the proper support that you need.” (Participant 2, Woman, 34 years, White).

## Discussion

Overall, service users had positive experiences associated with remote symptom-triggered alcohol withdrawal management and some participants reported they would not have otherwise accessed alcohol withdrawal management. As part of the benefits of being in the home environment, participants reported increased comfort, privacy, flexibility to engage in other activities, normalizing abstinence in the home and convenience. Participants also found the frequent interactions with staff, accountability, and use of counselling and MAUD helpful. Areas for improvement included prescribing medication in advance to prevent delays in medication pickup, enhancing preparation prior to the intervention, and providing more aftercare services.

The results of this study are consistent with past research assessing service user experience of telemedicine-delivered mental health interventions generally [2, 3, 5, 18], and in AUD specifically [9, 19], which have found positive client experiences. Consistent with past research, some participants reported difficulty with non-verbal cues [20] or desire for physical examination [7, 21]. However, overall, it seems that the benefits of the home environment, specifically convenience, flexibility, and comfort [2, 7, 9] outweighed the technological tensions arising from not having a face-to-face encounter.

The convenience of not travelling to a clinic or hospital that is associated with telemedicine has been described as telemedicine’s “erasure of distance” [22], and is often evoked, as in the rationale of the development of this intervention, to facilitate access to healthcare in regions without access to specialized services. Rather than erasure of place however, what participants described was that space itself (the home environment) was reshaped. As Oudshoorn (2012) describes, “technologies participate in redefining the meaning and practices of spaces in which they are used.” This redefining of place has been termed the ‘technogeography’ of care and describes how “[technologies change] the landscape of care by connecting previously distinct places, redefining the meaning of these places, and creating new sites where care takes place [23]”. Participants in our study reported the experience of remote withdrawal management in the home re-cast their home as possible spaces for health, as spaces they could newly imagine being sober in and gave them confidence to continue seeing the home as a positive space for health even beyond the remote intervention. This suggests possible benefits of remote alcohol withdrawal management beyond what had originally been imagined and has implications for remote delivery of other alcohol use disorder interventions such as virtual ‘residential treatment’.

The highlighted areas for improvement are feasible with minor refinements to the intervention. Specifically, during the intervention, procedures were changed to allow pick-up of medication the day before withdrawal management started, which alleviated concerns with respect to the logistics of acquiring the medication. Similarly, embedding remote withdrawal management within existing clinical workflows could reduce barriers to scheduling and reduce wait times for treatment as delays in scheduling were related to required research procedures. Attention to timely integration into existing aftercare programs could improve service user support following completion of withdrawal management. Participants also made suggestions with respect to improved preparation for the procedure, although there was not consensus about how it might be improved. This may suggest that individuals require tailored preparation.

Within the intervention-specific feedback, participants’ perception of the role and meaning of support persons were diverse. Interestingly, the Canadian alcohol use disorder guidelines recommend the availability of a support person “who can monitor symptoms during the acute withdrawal period (i.e., 3–5 days) and support adherence to medications,” as a criterion for outpatient withdrawal management [24]. The ASAM clinical guideline on alcohol withdrawal management also suggests taking into consideration the availability of social support in determining the level of care needed for withdrawal management [25]. The varying service user perspectives on the benefits and risks of support persons suggests their use should be evaluated prospectively for non-monitored and monitored home withdrawal management to understand if the assumptions about their utility with respect to safety or positive service user outcomes are correct. It was not possible with our small sample size to make comparisons based on participant or support person age/gender or treatment history and future research should consider how these characteristics impact service user outcomes. Based on the disparate experiences of our participants, including those who found it unnecessary and those who found it to be harmful, our findings suggest that the lack of a support person in the home should not inherently be a barrier to home-based alcohol withdrawal management, and that an individualized approach may be warranted.

We note that this intervention is being promoted within an era of “technological triumphalism” [26] and “promissory discourse” [27, 28] surrounding digital mental health interventions, wherein telemedicine applications are presented as a “technological fix” [27] amid crises of rising healthcare costs, human resource shortages, and fragmented mental health systems. Indeed, the fragmentation of substance use treatment systems has been previously described, and includes concerns about limited capacity, long waitlists, lack of service coordination, and difficulty with transition between withdrawal management and residential treatment [29, 30]. Although digital technologies have been posited as a way to improve efficiencies of the system, constrain cost, increase convenience, and improve access, our interviews highlight participants’ persistent difficulties in navigating a complex and under-funded mental healthcare system. This was evident in areas participants liked most about the intervention (interactions with staff), as well as what they perceived to have gained from participation (connections to other programs or ongoing care), their desires for more robust aftercare, and their complaints about difficulty navigating a fragmented system. From a practical perspective, it highlights the role of improving care coordination. This might be accomplished by building interventions with structured referral and care pathways, robust connections to ongoing care, or utilizing peer support and case management to enable seamless transition across services [31–33].

It has been well documented that innovations in medicine can face long delays from the time of development to widespread adoption [34, 35]. The main implementation challenges we foresee with respect to adoption of remote alcohol withdrawal management are (1) translating evidence such that practitioners feel comfortable treating service users remotely rather than in-person (2), ensuring that the intervention is being delivered with fidelity (e.g., service users are being selected appropriately and receiving an adequate number of daily check-ins), and (3) making sure treatment is adequately tailored to the communities where it is being delivered (e.g., the treatment is adapted so that it leverages community resources and is culturally appropriate). Future directions for research that would support implementation may include a larger study comparing unmonitored home versus supervised remote alcohol withdrawal management, research describing local adaptations of the intervention, research investigating in what situations support-persons improve outcomes, and research investigating service user experiences across diverse settings/populations.

### Limitations

The study has limitations. Equity concerns related to access and use of video telemedicine-based interventions for people who use substances has previously been raised, particularly in persons who are marginally housed, have severe mental illness, or lack access to computers or phones [3, 36–38]. Our study design only included individuals who were enrolled in the parent study where houselessness was an exclusion. Nevertheless, efforts to include participants broadly were developed for the parent study including making remote-enabled tablets available to participants who did not have a device with internet connectivity and including persons with concurrent mental illness unless they were acutely unstable, increasing the generalizability of our findings. Secondly, we considered only service user perspectives, and did not include support person perspectives or healthcare provider perspectives, which may have provided additional information to support intervention refinement.

## Conclusion

Service users perceived remote alcohol withdrawal management to be satisfactory and associated with several benefits including increased comfort, privacy and security, normalization of abstinence in the home, flexibility, and convenience. Service users also provided important feedback for refinement of the intervention, including improved preparation, embedding remote withdrawal within existing care pathways, and seamless pickup of medications. Future research should seek to explore how remote alcohol withdrawal reshapes the home as a care environment, whether and for whom inclusion of a support person improves outcomes, and compare remote symptom-triggered alcohol withdrawal management with monitored ambulatory alcohol withdrawal management and/or non-monitored at-home withdrawal management. Overall, findings suggest that remote alcohol withdrawal management could play an important role in improving access to medically-supervised alcohol withdrawal management, particularly in rural, remote, or underserved areas.

## Electronic supplementary material

Below is the link to the electronic supplementary material.

Supplementary Material 1

## Data Availability

De-identified data that support the findings of the study may be made available for sharing upon reasonable request.
