# Supplementary material for: “It beats the hell out of going to a hospital”: service user experiences of telemedicine-based symptom-triggered alcohol withdrawal management
Source: Addict Sci Clin Pract. 2025 Aug 13;20:68. doi: 10.1186/s13722-025-00585-8 (PMC12345073; doi:10.1186/s13722-025-00585-8)
Supplement: Supplementary file 1 — Supplementary Material 1 [file 13722_2025_585_MOESM1_ESM.docx]

Appendix 1: Interview guide – Service user interviews

| **Topic** | **Interview questions** |
| --- | --- |
| Introduction/Welcome | Review aim of study and purpose of research |
| Experience with remote alcohol withdrawal management | Can you describe how you became involved in the remote alcohol withdrawal management study?  🡪 What motivated you to participate? |
|  | How did you feel about your experience participating in remote alcohol withdrawal management?  🡪 What were the challenges |
|  | Did you have a support person with you? What role did the support person play?  If applicable: 🡪Can you give me an example of how they did or did not help you through the process of withdrawal?  🡪Do you think this role is needed? |
|  | What was one of the best things about the remote withdrawal experience? |
| Experience with telemedicine technology | What was it like using the telemedicine technology?  🡪How did you feel when using the technology? |
|  | What was your experience of communication with the study team? |
| Impact of treatment | What if anything has changed for you as a result remote alcohol withdrawal management?  🡪Was the experience easy, convenient, cumbersome? |
| Suggestions | What could be improved? |
|  | Is there anything that could have better prepared you for remote alcohol withdrawal management? |
|  | If you were to speak with someone considering this treatment, what would you tell them? |
| Final thoughts | Is there anything else you would like to add about your experience? |
